# Beyond BMI: waist circumference and social environment is associated with motor performance ability in kindergartners

**DOI:** 10.1186/s12887-019-1872-1

**Published:** 2020-01-06

**Authors:** Sascha W. Hoffmann, Matthias Dreher, Michael S. Urschitz, Perikles Simon

**Affiliations:** 1 Department of Sports Medicine, Disease Prevention and Rehabilitation, Faculty of Social Science, Media and Sport, Johannes Gutenberg-University Mainz, Albert-Schweitzer-Straße, 22, 55128 Mainz, Germany; 2 Department of Sports Medicine / Sports Physiology, Institute of Sport Science, University of Bayreuth, Universitätsstraße 30, 95440 Bayreuth, Germany; 3 Division of Pediatric Epidemiology, Institute of Medical Biostatistics, Epidemiology and Informatics, University Medical Center of the Johannes Gutenberg University Mainz, Obere Zahlbacher Straße 69, 55131 Mainz, Germany

**Keywords:** Overweight, Obesity, Kindergartners, Body mass index, Waist circumference, Social environment, City District

## Abstract

**Background:**

The aim of the current study was to examine the relationship between anthropometric characteristics (i.e. body height, body weight, body mass index [BMI] and waist circumference [WC]) with motor performance ability [MPA], social environmental factors of the district (i.e. employment status/working life, education, social situation/heterogeneity and home environment), where the respective kindergarten was located, as well as other potential health determinants in a representative sample of kindergartners.

**Methods:**

We analyzed data of 434 children aged 3 to 6 years which were obtained from a community-based cross-sectional health study conducted in the city of Mainz, Germany. Body height and weight, BMI and WC standard deviation scores [SDS] were calculated relative to the international proposed cut-offs of the IOTF. MPA was collected with multiple test items to determine coordination, speed strength, muscular endurance and speed. The life situation index [LSI] was used to assess the social environment of the district of the kindergarten. Adjusted for covariates, correlation and logistic regression analyses were conducted to estimate the effect of WC on MPA.

**Results:**

Below-average MPA was found in 46% of the sample. While there was no relationship to BMI (odds ratio [OR]: 1.09, 95% confidence interval [95% CI]: 0.83–1.44; *p* = 0.538), WC SDS was positively associated with below-average MPA (OR: 1.41, 95% CI: 1.01–1.95; *p* = 0.041). Further results show that the social environment of the district of the kindergarten was independently related to below-average MPA (OR: 2.72, 95% CI: 1.29–5.75; *p* = 0.009).

**Conclusion:**

The findings suggest that WC rather than BMI is linked to measurements of MPA already in kindergartners and furthermore, there seems to be an independent association between MPA and the social environment of the district of the respective kindergarten.

## Background

The increase of overweight/obesity in childhood is a major public health concern [1–3]. The worldwide estimated prevalence of overweight/obesity in children in 2010 was 6.7% and is expected to reach an estimated 9.1% in 2020, which would affect about 60 million children [4]. Depending on the country, the prevalence might be much higher. For example, 23% of Australian preschoolers between 2 and 4 years are overweight/obese and boys in the age of 5–7 year show the highest obesity rate [5]. Between 1975 and 2016 global age-standardized mean body mass index (BMI) in children and adolescents aged 5–19 years varied widely by region and decade, while BMI increased gradually over the four decades of analysis. However, a recent flattening of trends was observed in northwestern Europe and the high-income English-speaking Asian-Pacific region for both genders; in southwestern Europe for boys and in central and Andean Latin America for girls [6].

The reasons for being overweight or obese in childhood are very diverse. On the one hand, the well-known risk factors like low physical activity, high sedentary activity, high screen time activity, unhealthy diet, inadequate sleep, psychological factors and a disadvantageous built environment have been widely studied, summarizing an increased risk of the development of overweight/obesity [7–11]. On the other hand, additive influence factors like the social environment, living situations, demography, budgetary structure, income maintenance, marginalized social groups, social structures in general, and educational aspects are less studied but play a potential role in shaping overweight/obesity risk [8, 12, 13].

Apart from the increase in childhood overweight/obesity, motor performance ability (MPA) deficits in children have significantly increased during the last decades, even in Germany [14–21]. MPA has deteriorated by an average of 10% [22]. Research suggests that childhood obesity may lead to impaired cognitive and physical development which could trigger a cycle of physical activity avoidance and reduced social interactions in later childhood [23]. Prior research on the association between obesity and impaired MPA shows conflicting results. While some studies and a systematic review [24] found impaired MPA in overweight/obese children independent of gender [25–27], other studies just showed reduced MPA amongst obese boys compared to non-obese boys [28, 29]. National representative data of MPA in children was provided by the „Motorik-Modul” (MoMo) conducted as a sub-survey of the German Health and Examination Survey of Children and Adolescents (KiGGS) [18, 20, 30]. Among the 4–11-year old children, girls showed a slightly higher motor fitness than boys. Additionally, the results indicated an association between motor fitness, migration background and socio-economic status [30]. Higher fundamental movement skills in boys compared to girls are well documented [31]. Overall, the results emphasize the relevance of an early focus on motor ability improvement to encourage overweight/obese children to be physically active [25]. The protective effect of fitness and motor competence to avoid overweight/obesity has already been shown [32].

However, tailored prevention strategies to improve children’s health and motor ability performance will be more effective, if representative community-based data is available. Although such data has been assessed in different parts of Germany; no data is available for the state of Rhineland-Palatinate [15, 16, 26, 33–38]. In addition, little is known about the relationship between anthropometric measurements and MPA at pre-school age [16] and published data was only related to BMI [25–27, 39, 40]. A systematic review reported only one article which assessed waist circumference (WC) in relation to fundamental motor skills [41]. In recent years, research suggests using other overweight-related variables rather than BMI for the prognosis of cardiovascular outcomes. BMI was shown to provide lower predictive ability and is seriously flawed, because it does not distinguish fat mass from fat-free mass [42, 43]. WC, in contrast, seems to be a better predictor of abdominal obesity in children [44]. Considering recent research, the main aim of the present study, was to analyze whether WC is better associated with MPA in kindergartners than BMI.

## Methods

### Data source

The present study is based on data of the Children Health Study of Mainz (CHSM) [45]. Parents of children attending one out of 34 of the 35 regional public kindergartens were informed and invited to participate in the CHSM. The purpose of the study was to collect data about health determinants associated with overweight/obesity in pre-school children and their related caregivers [45]. Children were also invited to participate in assessments of anthropometry measurements and MPA tests.

Of 869 parents contacted, 558 replied to our invitation and filled out a questionnaire. Six children were excluded because they lived with their grandparents, foster or adoptive parents. A further 81 were excluded due to incomplete information about body height and weight and their habitual physical activity scores. In addition, thirty-seven children were excluded due to inability to perform the MPA tests (*n* = 35) and the absence of WC measurements (*n* = 2). Thus, complete data was available from 434 parent-child pairs. For inclusion in the present study, children had to be free from acute and infectious diseases, chronic diseases and restrictions related to the active and passive musculoskeletal system. A detailed description of the active recruitment strategy is presented elsewhere [45]. Participation in this study was voluntary and all parents of participants provided written informed consent. To increase the level of anonymity, personal data collection was reduced to a minimum.

### Questionnaire

One of the child’s primary guardians completed a self-constructed standardized paper and pencil questionnaire. During informative meetings about the aims and procedures of the study in each kindergarten, parents were informed about the MPA screening of their child, about the health benefits of regularly conducted physical activity, especially in the family environment, and furthermore about the potential early life determinants of overweight/obesity [3]. Essentially, the questionnaire consisted of two main parts. The first part asked about children’s sociodemographic and anthropometric data like age, gender, height and weight at birth as well as questions about the current health status and language which is primarily spoken by the child and the parents. According to Rapp et al. [46], participants responded when they picked up their child from kindergarten to calculate daily time children attended the kindergarten. Additional questions were asked about physical activity habits and screen time activity (characterized with daily TV time and using the PC/Internet on weekdays/weekends) [47, 48]. Children’s physical activity level was assessed using a modified Baecke Habitual Physical Activity Questionnaire (HPAQ) [49] in which parents reported about their child’s physical activity behavior [50, 51]. Questions about screen time activity have already been used in the German Health Interview and Examination Survey for Children and Adolescents (KiGGS) [48, 52]. Detailed information of the raised questions and all classifications regarding habitual physical activity level and screen time activity were presented in a previous publication [45].

The second part focused on parental anthropometric characteristics (age, gender, weight, height), cultural background (country of birth, nationality), educational and employment level, alcohol intake, and smoking status. Parental BMI was calculated according to the WHO criteria [53] and education level was used to represent the individual socio-economic position (SEP) and was categorized into three levels: low educational level (primary school or none), middle educational level (vocational secondary modern school or equivalent), as well as high educational level (university or vocational postsecondary school) [54].

### Assessment of anthropometric measurements in children and definition of overweight/obesity

Children’s height and weight was measured by trained staff with a calibrated portable stadiometer (SECA 217 [SECA, Hamburg, Germany]) and with a calibrated flat scale (SECA 803). Children were weighed without shoes and wore light clothes. WC was measured according to proposed international proceedings [55–58] using an inelastic tape (SECA 203).

Overweight/obesity were defined according to the IOTF international proposed age- and gender-specific cut-offs. The IOTF cut-offs and international thinness cut-offs are used to classify children aged 2–18 years as thin, normal weight, overweight or obese, based on adult BMI cut-offs at 18 years [59]. BMI and WC SDS were calculated according to the international commonly used UK 1990 reference [60–62]. WC risk groups were categorized using German WC reference values obtained in 3–11 years old children [57]. Due to the fact that WC is a highly sensitive marker for abdominal obesity in childhood [44, 57, 63] and other anthropometric characteristics (i.e. weight, height, BMI) tend to underestimate obesity in youth [64], two modified risk groups in accordance with Schwandt et al. [57] were used: (1) No risk (<90th percentile) and (2) High risk of having multiple cardio-metabolic risk factors (MCRF; 90th - <97th percentile).

### *Assessment of* motor performance ability [MPA]

MPA of coordination, speed strength, muscular endurance and speed was assessed with multiple test items (One leg stand, standing long jump, lateral jump) of the modified Karlsruher Motor Ability Screening Test (KMS 3–6) [35], the Motorik Modul of the KiGGS-Study [18] and the KiMo project [16]. Reliability and validity of the test items (r ranging from 0.8 to 0.9) have been shown previously [35].

The following criteria have been applied for selection of test items [15]:

- Economy of feasibility
- Greatest possible acceptability by study participants
- Correlative relationships with health-related questions

Additionally, the “shuttle run” test, already conducted by Krombholz in 2004 [36], was employed to depict the MPA of speed. Due to the possibility of methodical difficulties which may arise in carrying out the “one leg stand” on a bar with a width of 3.0 cm at pre-school age, one leg stand was performed on a bar with a width of 4.5 cm in accordance with Krombholz [36] and De Toia et al. [16]. All test results were classified according to the summarized age and gender-specific reference values [16, 35, 36]. Due to the fact that there were no significant differences between normal weight and overweight/obese children in the test item “sit and reach” in the proposed age groups [20], this test item was omitted.

MPA tests were conducted in each kindergarten during mid-morning and each child was instructed by one trained member of the staff.

A detailed description of the test items with information about material, test construction and realization was published elsewhere [65]. Data collection generally took 10–12 min per child and each staff member demonstrated the tests before the child performed the test. There was no special order for the test items and up to four children performed the tests simultaneously, depending on the respective spatial conditions in the test room.

### Social environment of the district of the kindergarten

Regarding the social environment, we examined if kindergartners who attended kindergartens which are in socially deprived city districts were more likely to be overweight or obese. As part of a social city district analysis conducted by Pfeiffer et al., [66] comprehensive information about general conditions (land use, current living situation, demography, budgetary structure, employment status of the family and income maintenance) and social structures (highly problematic family status and educational level) were collected from each city district of the city of Mainz. This led to the computation of a Life Situation Index (LSI) based on employment status/working life, education, social situation/heterogeneity and home environment, summarized to one scale and which we used in this study. Detailed description and method of calculation for demonstrating socio-demographic and socio-economic differences are presented in our previous studies [45, 47].

### Statistical analysis

Descriptive anthropometric characteristics were presented as means, frequencies and percentages with standard deviations (SD), respectively. The results of the MPA test items of the children were calculated for the total sample for gender, BMI groups, WC risk groups and LSI groups and were presented as medians and interquartile range (IQR). The software package LMSgrowth, Version 2.77, was used to calculate age and gender-specific standard deviation scores of height, weight, BMI and WC. For details of the methods we refer to previous publications [59, 67, 68].

For the MPA test results, we conducted group comparisons between boys and girls and normal weight and at least overweight children, as well as between WC risk groups and the LSI groups of each kindergarten using the Mann-Whitney U-Test.

In addition, descriptive characteristics of the final parental reports were employed and stratified by gender. Product-moment correlation coefficients, adjusted for age, BMI SDS and WC SDS, migration background, LSI and parental BMI were calculated to analyze the relationship between the MPA test items and the indices of children’s physical activity and investigate the associations between the indices of children’s physical activity and the indices of physical activity of their parents.

The association between potential determinants and MPA was examined using multiple logistic regression analysis and odds ratios (ORs) and the corresponding 95% confidence intervals (95% CI) were calculated. Continuous variables were categorized. JMP 8.0 (SAS, Cary, NC) and SPSS PASW 22 Statistics (IBM Corp., Somers, NY) were used for statistical analyses. Overall power analyses indicated a power ranging from 97 to 99% with *p*-values below 0.05.

## Results

### Sample characteristics

Table 1 presents the descriptive characteristics of the study sample. Mean age (± SD) was 4.9 ± 1.0 years and 55.9% were boys. Girls were significantly older (*p* = 0.009) than boys, whereas BMI was higher in boys (*p* = 0.043). Prevalence of overweight/obesity was significantly higher in boys than in girls (*p* = 0.027). In summary and according to the IOTF cutoffs, 18.1% were overweight/obese.

**Table 1:** Descriptive characteristics (Mean ± SD^a^) of the total study sample stratified by gender

Item	Boys	Girls	Total
N	243	191	434
Age	4.8 ± 1.0	5.0 ± 1.0	4.9 ± 1.0
Height, cm	109.8 ± 7.8	110.5 ± 8.5	110.1 ± 8.3
Height SDS	0.5 ± 1.1	0.4 ± 1.0	0.4 ± 1.0
Weight, kg	19.6 ± 3.8	19.4 ± 3.5	19.5 ± 3.7
Weight SDS	0.5 ± 1.1	0.3 ± 0.9	0.4 ± 1.0
BMI, kg/m^2^	16.2 ± 1.6	15.8 ± 1.4	16.0 ± 1.5
BMI SDS	0.3 ± 1.2	0.1 ± 0.9	0.2 ± 1.0
Waist circumference, cm	53.3 ± 4.2	52.6 ± 3.6	53.0 ± 4.0
Waist circumference SDS	0.4 ± 1.1	0.4 ± 0.9	0.4 ± 1.0
Thinness grade I, % (n)^b^	4.9 (12)	6.3 (12)	5.5 (24)
Normal weight, % (n)	72.8 (177)	81.2 (155)	76.5 (332)
Overweight, % (n)	15.6 (38)	11.0 (21)	13.7 (59)
Obese, % (n)	6.6 (16)	1.6 (3)	4.4 (19)

^a^SD = Standard deviation

^b^Thinness grade I-III were combined into one single thinness grade I due to small sample size

Total available parental characteristics concerning age, body height, body weight, calculated BMI with respective BMI groups, smoking status, country of birth, nationality and SEP from the questionnaire by males and females are presented elsewhere [45]. Results of the final parental sample descriptions of the items of habitual physical activity, screen time activity as well as the overweight/obesity status and the perception of weight status and associated health risks stratified by gender are also presented elsewhere [45]. The majority of parents analyzed in the final sample (*n* = 434) were female (85.7%). Indices of habitual physical activity did not differ between genders. Prevalence of overweight was higher in responding fathers compared to mothers (53.3 to 35.4%; *p* = 0.008). Moreover, responding fathers had higher PC/Internet use on weekends than mothers (*p* = 0.007).

### Motor performance ability [MPA]

**Fig. 1:**
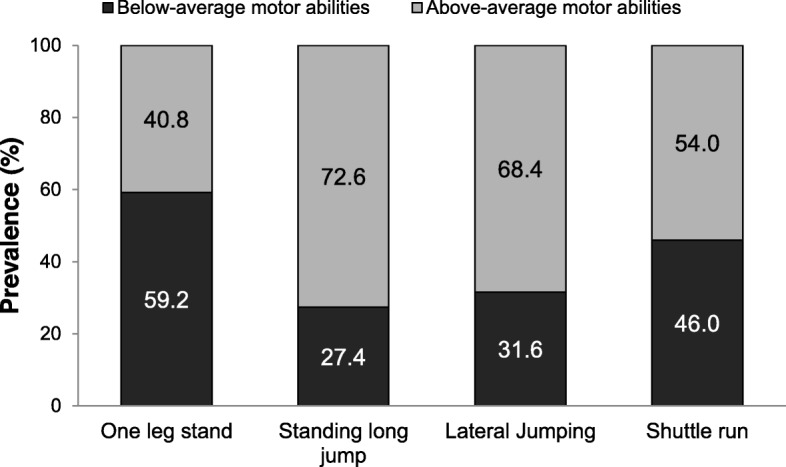
Relative frequencies of below-average and above-average motor ability in kindergartners aged 3 to 6-years

Below-average MPA was found in 46% of the total sample, while this was present in 59.2% of the children for the one leg stand, in 27.4% of the children for the standing long jump, in 31.6% of the children for lateral jumping, and finally in 46.0% of the children for the shuttle run test (see Fig. 1).

As shown in Table 2, girls had significantly less floor contacts than boys in the test item “one leg stand” (*P* < 0.001). No differences were found in the test items “standing long jump”, “lateral jumping” and “shuttle run”. Regarding the relationship of MPA and BMI status, Table 3 shows that significant BMI group differences were only observed within the test item “lateral jumping” between children classified into normal weight and overweight/obesity. Overweight/obese children performed significantly less jumps than children in the normal weight group (21.6 ± 12.1 vs. 29.4 ± 12.2; *p* = 0.039).

**Table 2:** Motor performance ability test items (Median and IQR^a^) of the total study sample stratified by gender

Test items	Median (IQR)	P^*^
One leg stand (floor contacts; n = 434)		
Boys	21.0 (10.0 to 30.0)	**< 0.001**
Girls	13.0 (7.0 to 30.0)	
Total	17.0 (7.0 to 30.0)	
Standing long jump (cm; n = 434)		
Boys	84.0 (63.0 to 103.0)	0.929
Girls	85.0 (67.0 to 103.0)	
Total	85.0 (65.0 to 103.0)	
Lateral jumping (jumps; n = 434)		
Boys	23.0 (18.0 to 30.0)	0.156
Girls	25.0 (19.0 to 31.0)	
Total	24.0 (18.0 to 31.0)	
Shuttle run (s; *n* = 424)		
Boys	10.5 (9.1 to 12.1)	0.383
Girls	10.6 (9.5 to 12.3)	
Total	10.6 (9.3 to 12.2)	

^*^P < 0.05; Mann-Whitney U-Test for gender differences; significance printed in bold type

^a^IQR = Interquartile range

**Table 3:** Motor performance ability test items (Median and IQR^a^) of the total study sample stratified by BMI^b^ group (IOTF^c^ cutoffs)

Test items	n	Median (IQR)	P^*^
One leg stand, floor contacts			
Normal weight	348	16.0 (7.0 to 30.0)	0.175
Overweight/obesity	76	17.5 (9.0 to 30.0)	
Standing long jump, cm			
Normal weight	348	86.5 (68.0 to 103.8)	0.513
Overweight/obesity	76	83.0 (65.0 to 102.6)	
Lateral jumping, jumps			
Normal weight	348	25.0 (19.0 to 31.0)	**0.039**
Overweight/obesity	76	22.0 (14.0 to 29.0)	
Shuttle run, s			
Normal weight	348	10.5 (9.3 to 12.3)	0.904
Overweight/obesity	76	10.7 (9.2 to 12.0)	

*P < 0.05; Mann-Whitney U-Test of motor ability performance test items between the modified BMI groups; significance printed in bold type

^a^IQR = Interquartile range

^b^BMI=Body mass index

^c^IOTF=International Obesity Task Force

In contrast to this, significant differences between the no risk WC group and the group with high risk of having multiple cardio-metabolic risk factors in all test items except in the “shuttle run” were observed (see Table 4).

**Table 4:** Motor performance ability test items (Median and IQR^a^) of the total study sample stratified by age and gender specific WC^b^ groups^c^

Test items	n	Median (IQR)	P^*^
One leg stand, floor contacts			
No risk <(90th percentile)	375	16.0 (7.0 to 30.0)	**0.014**
High risk of having MCRF (90th - <97th percentile)	49	26.0 (11.5 to 30.0)	
Standing long jump, cm			
No risk <(90th percentile)	375	87.0 (69.0 to 105.0)	**0.008**
High risk of having MCRF (90th - <97th percentile)	49	76.0 (56.0 to 91.0)	
Lateral jumping, jumps			
No risk <(90th percentile)	375	25.0 (19.0 to 31.0)	**0.003**
High risk of having MCRF (90th - <97th percentile)	49	20.0 (14.5 to 25.5)	
Shuttle run, s			
No risk <(90th percentile)	375	10.5 (9.3 to 12.2)	0.126
High risk of having MCRF (90th - <97th percentile)	49	11.1 (10.0 to 12.1)	

^*^P < 0.05; Mann-Whitney U-Test of motor ability performance test items between the different WC groups; significance printed in bold type

^a^IQR = Interquartile range

^b^WC = waist circumference

^c^Modified WC groups were conducted according to Schandt et al. (2008)

Table 5 shows the relationship of motor ability performance and the social environment of the district of the kindergarten expressed as two LSI groups. Significant group differences between LSI groups were observed in the test items “one leg stand” (*p* = 0.044) and “standing long jump” (*p* = 0.043).

**Table 5:** Motor performance ability test items (Median and IQR^a^) of the total study sample stratified by the social environment of the district of the kindergarten (LSI^b^ groups)

Test items	n	Median (IQR)	P^*^
One leg stand, floor contacts			
LSI low	175	17.0 (9.0 to 30.0)	**0.044**
LSI high	249	15.0 (7.0 to 30.0)	
Standing long jump, cm			
LSI low	175	83.0 (65.0 to 100.0)	**0.043**
LSI high	249	89.0 (67.5 to 106.0)	
Lateral jumping, jumps			
LSI low	175	24.0 (19.0 to 30.0)	0.726
LSI high	249	24.0 (18.0 to 31.0)	
Shuttle run, s			
LSI low	175	10.9 (9.6 to 12.2)	0.129
LSI high	249	10.4 (9.2 to 12.1)	

^*^P < 0.05; Mann-Whitney U-Test of motor ability performance test items between the LSI groups; significance printed in bold type

^a^IQR = Interquartile range

^b^LSI is defined by Life Situation Index scores (based on employment/working life; education; social situation/heterogeneity and home environment) according to Pfeiffer et al. (2005); score ranges from −10 to + 10 with 0 as median

Determinants of below-average motor ability from multiple regression analysis are shown in Table 6. The final regression model indicated that BMI was not integrated in the final model and it seems that in kindergartners WC is a better predictor of motor ability than BMI. Analyses furthermore indicated that older children performed better in motor ability tests than their younger counterparts; hence, younger children were at higher risk for below-average motor ability than older children. Additionally, the social environment of the district of the kindergarten was significantly positive related to below-average motor ability. Finally, there were additional positive associations with parental sport index as well as with children’s sport and leisure-time index (data not shown).

**Table 6:** Determinants of below-average motor performance ability in kindergartners aged 3–6 years^a^

Item	OR^b^	95% CI for OR^c^	P^*^
Children’s age			
3–4	**2.81**	**1.00–7.86**	**0.049**
5–6	1.00		
Parental sport index^d^			
Low	**2.07**	**1.18–3.63**	**0.011**
High	1.00		
Children’s sport index^d^			
Low	**3.24**	**1.85–5.67**	**< 0.001**
High	1.00		
Children’s leisure-time index^d^			
Low	**2.09**	**1.14–3.85**	**0.017**
High	1.00		
Children’s waist circumference SDS^e^	**1.41**	**1.01–1.95**	**0.041**
Social environment (LSI) of the district of the kindergarten^f^			
Low	**2.72**	**1.29–5.75**	**0.009**
High	1.00		

^a^Only significant associations are displayed

^b^OR = Odds ratios

^c^OR and 95% confidence intervals (CI) are from multiple logistic regression analysis in which all independent variables were included simultaneously: Kindergarten teachers BMI, kindergarten teachers age, kindergarten teachers habitual physical activity, children’s gender, children’s age, children’s migration background, educational level, parental BMI, parental habitual physical activity, children’s body mass index standard deviation scores, social environment of the district of the kindergarten (LSI), children’s screen time activities, children’s physical activity level

^d^Sport index and leisure-time index was derived from the habitual physical activity score conducted by Baecke et al. (1982). Scores were dichotomized. The lower the score, the more likely the participant had lower physical activity levels

^e^Standard deviation scores according to the UK 1990 reference by Cole et al. (1998)

^f^LSI = Life situation index was conducted analogously to the study of Hoffmann et al. (2013)

^*^P ≤ 0.05

## Discussion

Results of the present study revealed determinants of below-average motor performance ability in a sample of 3–6-year-old kindergartners.

Significant determinants of below-average motor ability were being of younger age (3–4 years), low children’s leisure-time and sport index, low parental sport index, high WC and attending a kindergarten located in a disadvantaged city district. Interestingly, results indicated that WC is a better predictor of MPA than the well-established BMI in kindergartners. In contrast to previous studies [26, 35], no association was found between MPA and BMI, except for the test item “lateral jumping” (*p* = 0.039).

The current study findings indicated that there is a high variance for WC in this young age group, already. According to Schwandt et al. [57], the combination of having a low BMI and a high WC may cause an adverse health development. Epidemiological studies proposed to use WC measurements in clinical settings, already at pre-school age, as well as in clinical practice (i.e. medical health examinations) with older children [56, 69], to monitor abdominal obesity. As previously shown, WC is a good predictor of intra-abdominal fat distribution in children and adolescents [70]. Measurement of WC is easy and cost-effective and the measurement may provide decisive benefits in order to identify those children who seemingly have a higher risk to develop obesity-related diseases later in adulthood [69]. In addition, recent research provides evidence that high WC is associated with increased systolic blood pressure, low-density lipoprotein cholesterol and triacylglycerol and decreased high-density lipoprotein cholesterol [71].

The second main result of our study provided evidence that the social environment of the district of the kindergarten represented by the LSI was independently related to MPA and it seemed to be that there is an apparent influence by the kindergarten’s location. In a previous study, we have already shown the influence of the (occupational) social environment shaping the risk of obesity in kindergarten teachers in their work environment [47]. Other studies reported about the neighborhood environment mostly in school children [72–74]. We suppose that this social area-level deprivation has a greater impact on children’s health than previously assumed. This work provides evidence that together with WC and other child characteristics, the city’s district and therefore the social environment of the district of the kindergarten, possibly affect children’s motor performance ability which is linked to children’s health. The study from Finn et al. [75] showed similar results as our study and pointed out that more than half of the variance in daily physical activity, was predicted by the kindergarten. The implementation of physical activity interventions has to begin as early as possible [76]. Kindergartens with their specific environment seem to play an underestimated role in health promotion in young children [45].

The present study assessed physical activity levels with self-administered standardized questionnaires [49] to parents, who had to report on their as well as on their child’s specific physical activity levels. Adjusted for covariates, there was a significant correlation between children’s sport index and parental sport index, as well as between children’s leisure-time index and the corresponding parental index. Additionally, low parental HPA was a significant predictor of childhood overweight/obesity (data not shown). These findings are consistent with previous research provided by Taylor et al. [77], who found a positive relationship between parental activity and child’s activity (r = .17–.28). In addition, Eriksson et al. [78] found similar associations of parent-child physical activity relationships between 12-year-olds and their parents and proposed to focus on the family environment to promote physical activity in children. In particular, joint physical activity in parent-child pairs could have health benefits, especially for girls, older children, older parents and high income families [79].

Manipulative skills refer to motor skills involving an object. They are all about making certain movements to apply force in order to move objects. Common examples include throwing, catching, kicking, or striking. Noteworthy, the standardized testing battery we employed here is devoid of manipulative motor skill performance and as such selective in nature. This limits the generalizability of our findings with regard to motor performance. As expected, older children showed better performance in the MPA test items than younger children. Similar results were shown in the MoMo study [20, 30, 80]. Girls significantly performed better in the “one leg stand” than boys and 4–6-year-old children attending kindergartens in Mainz showed better results in the test items “one leg stand” and “lateral jumping” compared to children, who participated in the MoMo study. Furthermore, and in contrast to preliminary findings [65], the results of the “standing long jump” indicated no differences between BMI groups. This is in accordance with results of the KiMo project, presented by De Toia et al. [16].

The results of our study are limited due to the use of a self-reported questionnaire that may have led to a systematic bias towards the directions of more socially desirable answers for questions about physical activity and anthropometrics. Specifically, only one parent reported personal and children-specific physical activity and screen time, which may have led to some additional invalidity. Furthermore, our cross-sectional design limits our ability to draw conclusions about the direction of the associations and analysis of our logistic regression should not be regarded as a causal relationship between the dependent and the independent variable. Besides the statistical and administrative limitations it should be mentioned that the LSI was used to describe the social environment of the city district, where the kindergarten is located. Since the kindergarten is commonly located close to the place of residence, we cannot exclude that parental socio-economic factors are major contributors to the associations with the LSI. Even though the study focused on the social environment of the neighborhood around the kindergarten on child-specific influencing factors, potential confounding child specific variables like unhealthy diet, psychological factors or inadequate sleep were not assessed and should be considered in future studies.

## Conclusion

The current findings supported the association between MPA and physical activity in kindergartners. Physical activity scores of the parents and the child were positively associated with the child’s respective MPA. Although the correlation coefficients between the MPA items and the indices of physical activity in children were low, there were more profound differences between physically active children and those who were less physically active during their daily life. Furthermore, we found a significant association of WC and the social environment of the district of the kindergarten with MPA.

Our study suggests that in kindergartners 3- to 6-years of age, important influencing factors for below-average motor ability can be identified, already. Therefore, future studies may verify and target these factors to more effectively intervene in young children by promoting physical activity in order to prevent overweight/obesity as well as other negative consequences of physical inactivity.

## Data Availability

The data sets used and / or analyzed during the current study are available from the corresponding author on reasonable request.

## Abbreviations

ANOVA: Analysis of variance
BMI: Body Mass Index
CHSM: Children Health Study Mainz
HPA: Habitual Physical Activity
HPAQ: Habitual Physical Activity Questionnaire
IOTF: International Obesity Task Force
IRB: Institutional Review Board
KiGGS: German Health and Examination Survey of Children and Adolescents
LSI: Life situation index
MCRF: Multiple cardio-metabolic risk factors
MoMo: Motorik-Modul
MPA: Motor performance ability
OR: Odds ratio
SD: Standard deviation
SDS: Standard deviation scores
SEP: Socio-economic position
TV: Television
WC: Waist circumferences
